# Activation-Induced Cytidine Deaminase Expression in CD4^+^ T Cells is Associated with a Unique IL-10-Producing Subset that Increases with Age

**DOI:** 10.1371/journal.pone.0029141

**Published:** 2011-12-28

**Authors:** Hongyan Qin, Keiichiro Suzuki, Mikiyo Nakata, Shunsuke Chikuma, Nakako Izumi, Le Thi Huong, Mikako Maruya, Sidonia Fagarasan, Meinrad Busslinger, Tasuku Honjo, Hitoshi Nagaoka

**Affiliations:** 1 Department of Immunology and Genomic Medicine, Graduate School of Medicine, Kyoto University, Kyoto, Japan; 2 Laboratory for Mucosal Immunity, RIKEN, Yokohama, Japan; 3 Research Institute of Molecular Pathology, Vienna Biocenter, Vienna, Austria; South Texas Veterans Health Care System, United States of America

## Abstract

Activation-induced cytidine deaminase (AID), produced by the *Aicda* gene, is essential for the immunoglobulin gene (*Ig*) alterations that form immune memory. Using a Cre-mediated genetic system, we unexpectedly found CD4^+^ T cells that had expressed *Aicda* (exAID cells) as well as B cells. ExAID cells increased with age, reaching up to 25% of the CD4^+^ and B220^+^ cell populations. ExAID B cells remained IgM^+^, suggesting that class-switched memory B cells do not accumulate in the spleen. In T cells, AID was expressed in a subset that produced IFN-γ and IL-10 but little IL-4 or IL-17, and showed no evidence of genetic mutation. Interestingly, the endogenous *Aicda* expression in T cells was enhanced in the absence of B cells, indicating that the process is independent from the germinal center reaction. These results suggest that in addition to its roles in B cells, AID may have previously unappreciated roles in T-cell function or tumorigenesis.

## Introduction

Activation-induced cytidine deaminase (AID) is essential for somatic hypermutation (SHM) and class switch recombination (CSR), which diversify the immunoglobulin gene in activated peripheral B cells [1], [2]. SHM introduces point mutations in the *V* region exon, thus contributing to the antibody affinity maturation associated with cell selection. CSR is a region-specific DNA recombination that occurs between two switch regions located 5′ to each heavy chain constant (*C_H_*) region gene. This recombination replaces the *V_H_*-proximal *C_H_* gene with a downstream *C_H_* gene by looping out the intervening sequence, thereby generating isotype-switched antibodies without changing the antigen specificity [3].

Both CSR and SHM are initiated by AID that induces the target DNA cleavages [4], [5]. In addition to immunoglobulin (*Ig*) genes, a considerable number of non-*Ig* genes, including proto-oncogenes, can be attacked by AID [6]. Furthermore, a growing number of reports suggest that, because of its mutagenic activity, AID may be involved in tumorigenesis in both B and non-B cells [7]–[12]. Studies indicate that the incidence of lymphoma carrying a *Myc-Ig* translocation is drastically reduced in AID-deficient mice [7], [12]. In contrast, systemic AID overexpression in transgenic mice consistently results in T-cell lymphoma, as well as lung tumor, liver tumor, and B lymphoma, with lower frequencies [13]–[16]. Notably, tumorigenic hepatitis C virus or *H. pylori* infection can induce AID expression [10], [17]. AID thus appears to be genotoxic, and its expression must be tightly regulated.

We previously showed *in vitro* that *Aicda* expression is regulated by a balance between enhancers and silencers [18]. The silencers E2f and c-Myb strongly repress *Aicda* transcription in non-B cells and non-stimulated B cells. When B cells are stimulated, B-cell-specific (Pax5 and E2a) and stimulation-responsive (NF-κB, STAT6, Smd3/4, and C/EBP) enhancers act in concert to overcome the silencers, thereby turning on *Aicda* transcription [18]. This model explains the mechanism by which AID is restricted primarily to activated B cells, yet can be induced in non-B cells by strong environmental stimuli [18].

In fact, high AID levels are found only in germinal-center (GC) B cells, which actively undergo SHM and CSR [19]. Small amounts of AID have been found in immature B cells, although the amount of AID and the percentage of AID-positive immature B cells have not yet been accurately measured. Significant levels of SHM and CSR have been observed in these cells, suggesting that AID might be involved in antigen-independent immunoglobulin diversification in B cells at an early developmental stage [20]–[22]. In addition, involvement of AID in B cell central tolerance in both human and mouse was suggested by recent two publications [23], [24]. These observations imply that even subtle level of AID expression would have important role in regulation of immune system. Small amounts of AID have also been found in the mouse ovary and human testis [25], [26], although AID's function in germ cells is not well established, since AID-deficient mice reproduce without obvious genetic disorders.

AID is difficult to detect convincingly at low levels. To overcome this problem, we introduced a genetic marking system using bacteria artificial chromosome (BAC) transgenic mice carrying Cre-ires-hCD2 knocked into the *Aicda* locus [27]. We also crossed this mouse with a genetically marked reporter mouse. This system allowed us to examine both past and current AID expression by detecting two genetic markers. Since the marked cells can accumulate in a cell population, the detection sensitivity for AID-expression history can be enormously enhanced. Using this system, we unexpectedly found that AID is expressed in a considerable fraction of CD4^+^ memory T cells in mice maintained under specific pathogen-free conditions.

## Results

### The Aicda-cre system efficiently monitors Aicda expression

The *Aicda-cre* transgenic mouse carries a 190-kb BAC DNA containing the entire *Aicda* locus (Figure S1A) [27]. The *Aicda* coding region on the transgene was engineered to generate the human CD2 and Cre recombinase instead of AID; thus, current *Aicda* promoter activation can be visualized by hCD2 staining. In addition, by crossing these mice with Rosa26 reporter mice (R26R or Rosa-tdRFP), previous *Aicda* promoter activation can be visualized, because Cre irreversibly turns on LacZ or RFP reporter expression at the *Rosa26* locus (Rosa reporters) [28], [29].

**Figure 1:**
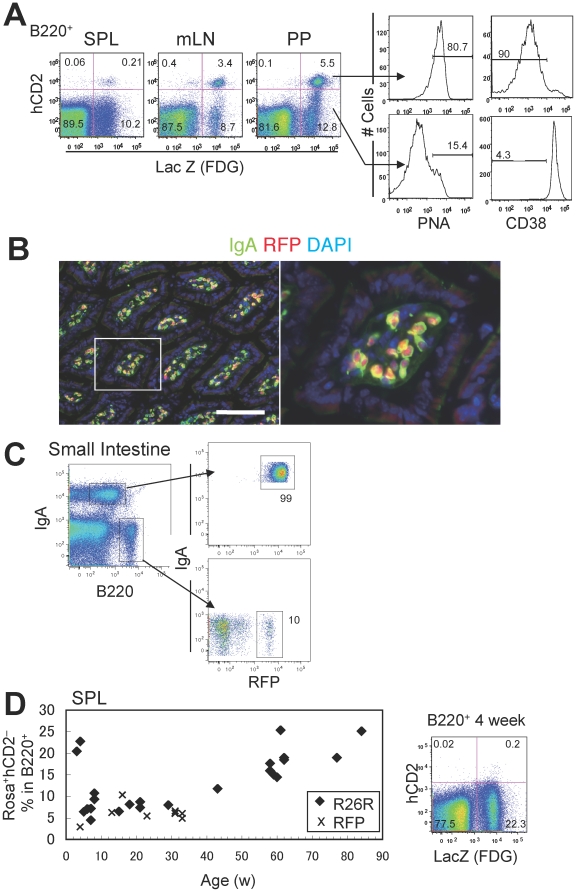
Aicda-cre/R26R or /Rosa-tdRFP B cells are efficiently marked upon their activation *in vivo*. (A) Peripheral lymphoid organs of Aicda-cre/R26R 21-week-old mice were stained with B220, human CD2, PNA, and CD38. Histograms show CD38 and PNA expressed in LacZ^+^ hCD2^+^ and LacZ^+^ hCD2^−^ cells. SPL, spleen; mLN, mesenteric lymph node; PP, Peyer's patch. (B) Immunofluorescence analysis of the small intestine of an Aicda-cre/Rosa-tdRFP mouse. IgA (green), RFP (red), and DAPI (blue) signals are shown together. The right panel shows an enlarged view of the area outlined in the left panel. Bar represents 100 µm. (C) FACS analysis of lamina propria cells from the small intestine of an Aicda-cre/Rosa-tdRFP mouse. (D) The percentage of B cells with the Rosa^+^ (LacZ or RFP) hCD2^−^ among B220^+^ cells in the spleen, plotted by age (in weeks); symbols indicate individual mice. Correlation coefficient (r) was 0.65. Human CD2 and LacZ expression in B220^+^ spleen cells from a 4-week-old Aicda-cre/R26R mouse are shown (rightmost panel).

We analyzed the Aicda-cre/R26R and Aicda-cre/Rosa-tdRFP mice and found substantial numbers of LacZ/RFP single-positive (exAID) and LacZ/RFP hCD2 double-positive B cells in secondary lymphoid organs (Figure 1A and Figure S1B). The majority of LacZ^+^hCD2^+^ B cells were peanut-agglutinin (PNA) positive, either CD38^−^ or CD95^+^, marking them as GC B cells (Figure 1A and Figure S1B); Rosa single-positives contained only a small fraction of GC B cells. This indicates that cells quickly lost AID expression after leaving the GC. Nonetheless, IgA^+^ plasmablasts, which should have expressed AID, were almost completely labeled by RFP in the Aicda-cre/Rosa-tdRFP mice (Figure 1B and 1C).

Initially, we thought the exAID B cells were post-GC cells. However, these cells comprised a substantial fraction of the B220^+^ cells in the spleen of young animals (3–4 weeks), although the percentage varied from 3–23% for unknown reasons (Figure 1D). The percentage of exAID B cells remained fairly stable at 5–10% between 5–40 weeks of age, after which it gradually increased, reaching a plateau of 20–25% (Figure 1D). The absolute numbers of exAID B cells followed a similar tendency; this was less apparent, however, perhaps due to the inevitable technical fluctuations of cell yield in each experiment (Figure S1C). The majority of accumulated exAID B cells in old animals remained IgM^+^IgD^+^, but SHM on the *IgV* downstream region was significant (Figure S1D and Table 1), suggesting that switched memory B cells may not accumulate in the spleen.

**Table 1:** Mutation frequency of *Jh4* downstream intron of *Igh* of B220^+^ CD3^−^ cells.

Cell	Mutation rate(/base pair)	Mutated base/Total base	Mutated clone/Total clone
LacZ^−^ hCD2^−^	1.0×10^−5^	1/96,000^a^^,^ b	1/160
LacZ^+^ hCD2^−^	3.0×10^−4^	30/98,400^a^	10/164
LacZ^+^ hCD2^+^	8.3×10^−4^	75/90,600^b^	8/151

*P* value (two tail Fisher's exact test):

a , p = 3.2×10^−8^;

b , p = 2.3×10^−22^.

Statistical comparisons between two types of cells (a- and b-pairs) were done by use of 2×2 contingency tables made by Mutated base/Total base of each pair.

**Figure 2:**
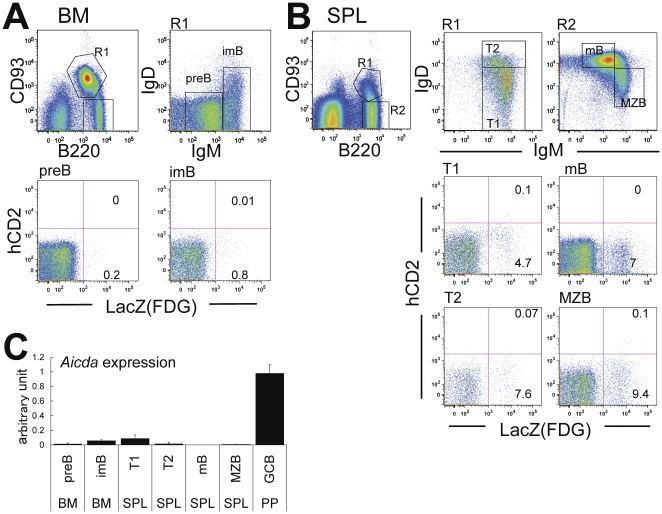
Low-level *Aicda* expression visualized in immature B cells. (A) Bone marrow (BM) cells of 7-week-old Aicda-cre/R26R mice were stained by the indicated markers. The corresponding gate for each panel is indicated. Numbers in each quadrant indicate the percentage of gated cells. (B) Aicda-cre/R26R spleen (SPL) cells were stained for the indicated markers. Gated areas are indicated in the top panels. (A)(B) Mean values and standard deviations of the percentage of LacZ+ cells in three 7∼8-week old mice were as following: preB = 0.33±0.16, imB = 1.03±0.23, T1 = 4.96±1.35, T2 = 7.74±2.99, mB = 7.96±2.69, MZB = 6.54±2.97. (C) *Aicda* expression is shown for each B-cell stage detected by RT-PCR; values were normalized to the *Gapdh* expression. Expression levels are presented relative to the average signal of germinal center B cells. The data represent the average of three independent experiments with standard deviation. R1, region 1; R2, region 2; preB, premature B cell; imB, immature B cell; T1, transitional B cell 1; T2, transitional B cell 2; mB, mature B cell; MZB, marginal zone B cell; GCB, germinal center B cell.

To determine if exAID cells could appear in a pre-GC population, we analyzed each stage of B-cell development. We found a few exAID cells among the immature B cells in bone marrow (Figure 2A and 2B). The fraction of exAID cells increased in the immature B cells in the periphery; transitional (T) 2 B cells included more than 7% exAID cells. We could not observe the hCD2 expression in immature cells by conventional hCD2 staining method, but the expression was confirmed by RT-PCR (Figure 2C) [21], [22], [30]. These results suggest that the exAID B-cell population found in the spleen may be, at least in part, generated in pre-GC stages. Taken together, the current Aicda-cre/Rosa-reporter system appeared to be sensitive to a much lower level of *Aicda* expression than could be detected by staining.

### ExAID cells in CD4^+^ T cells

**Figure 3:**
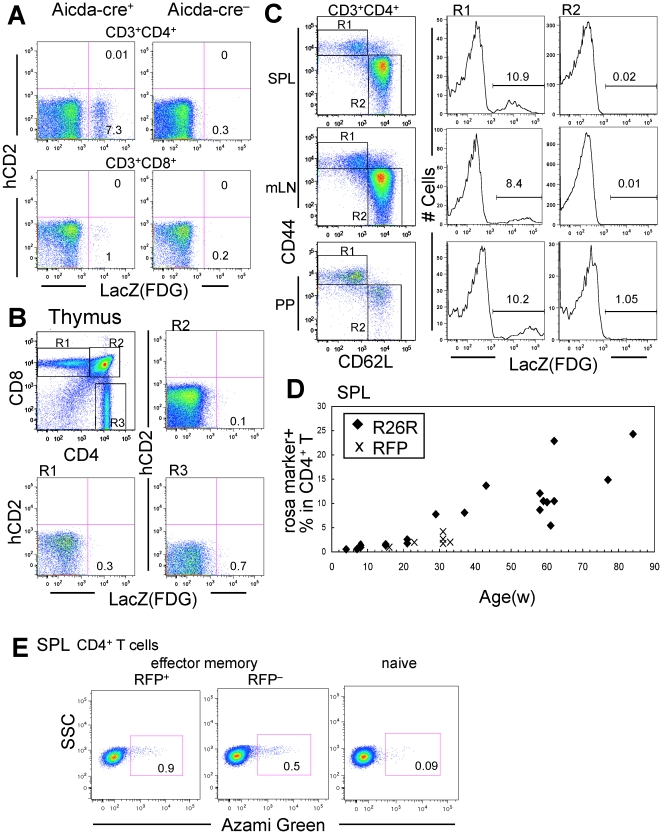
Aicda-cre/R26R mice reveal the history of *Aicda* expression in a subset of T cells. Spleen T cells (A), thymocytes (B), and peripheral lymphoid organs (C) from Aicda-cre/R26R mice were stained with the markers indicated. The gating used for each plot is shown at the top of each panel. Data shown is for 29-week-old (A and C) or 7-week-old (B) animals. Numbers in the panels show the percentage of gated cells of each quadrant/gate. (A) R26R (Aicda-cre^−^) is shown as a negative control. (C) R1 and R2 gating represents EM and naive T-cell populations, respectively. FACS data shown in (B) are representative of analyses of the thymus in three independent experiments, using mice aged 7, 15, and 18 weeks. (D) The percentage of LacZ^+^ (diamond) or RFP^+^ (cross) cells in CD4^+^ T cells in the spleen, plotted by age. Symbols represent individual mice. Correlation coefficient (r) was 0.86. (E) Aicda-cre/Rosa-tdRFP/Fucci mouse (64-week) spleen TCRβ^+^CD4^+^ cells were sorted into CD44^hi^ RFP^+^ and RFP^−^ EM cells as well as CD44^lo^ naïve cells. Cells were gated to the S/G2/M phase of the cell cycle. Results are representative of analyses of 4 animals. SPL, spleen; mLN, mesenteric lymph node; PP, Peyer's patch; R1, region 1; R2, region 2; R3, region 3.

Surprisingly, exAID cells also comprised about 7% of the CD3^+^CD4^+^ T cells in the spleen of 7-month-old Aicda-cre/R26R mice (Figure 3A and Figure S2AB). The frequency of exAID CD3^+^ CD8^+^ T cells was much lower than in CD3^+^CD4^+^ T cells. No clear hCD2^+^ T cells were observed, indicating that the *Aicda* expression in T cells is either very weak or rare at any given moment. ExAID CD4^+^ T cells had the effector memory (EM) phenotype (CD44^+^ CD62L^lo^) in the spleen [31], [32], lymph nodes and Peyer's patches, and few, if any, LacZ^+^ naïve T cells were observed (Figure 3C and Figure S2C). A small fraction of NK cells also expressed the *Rosa* marker (Figure S3A). In the thymus, exAID cells were not found in either CD4/CD8 double-positive or single-positive fractions (Figure 3B). Taken together, exAID T cells are likely to be generated in association with T-cell activation in the periphery. The percentage of LacZ^+^CD4^+^ T cells was consistently negligible in mice younger than 20 weeks, but gradually increased to around 25% of the total CD4^+^ T cells at around 18 months, although there were variations in individual animals (Figure 3D and Figure S3B). Furthermore, by crossing Aicda-cre/Rosa-tdRFP mice with Fucci transgenic mice, in which a cell-cycle indicator produces an Azami Green signal in the S/G2/M phase [33], we found that exAID CD4^+^ EM T cells proliferated as steadily as non-exAID EM T cells (Figure 3E). In the CD8^+^ (or CD4^−^) fraction there were generally very few exAID cells, which did not increase as quickly as those in CD4^+^ cells except in a couple of older animals (Figure S3C). It is thus likely that a certain fraction of activated CD4^+^ T cells, and a smaller but still detectable fraction of CD8^+^ T cells, can induce *Aicda* expression, and such exAID T cells can accumulate with age.

**Figure 4:**
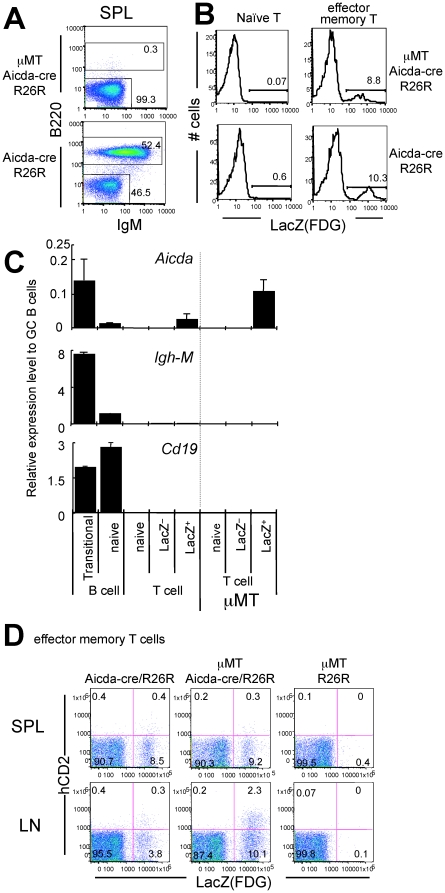
T-B interaction is not required for *Aicda* expression in T cells. (A) Results of B220 and IgM staining of Aicda-cre/R26R/μMT and Aicda-cre/R26R spleen (SPL) cells from 35-week-old animals are shown. (B) LacZ expressed in Aicda-cre/R26R/μMT (upper) and Aicda-cre/R26R (lower) spleen T cells. Naive and EM CD4^+^ T cells were gated as populations with CD3^+^CD4^+^CD62L^hi^CD44^lo^ and CD3^+^CD4^+^CD62L^lo^CD44^hi^ phenotypes, respectively. FACS data shown are representative of five independent experiments. (C) Endogenous *Aicda* mRNA expression in peripheral B and CD4^+^ T cells was quantified by real-time PCR with Taqman® probes. Pooled spleen and lymph node cells were sorted and analyzed. Each value was normalized to the *Ppib* signal and shown as a number relative to the signal of germinal center B cells in each experiment; therefore, in this graph, one corresponds to the signal equivalent to germinal center B cells. The error bar indicates the standard deviation of triplicate PCR. The results are representative of two independent sorting experiments using two animals each. The following markers were used to define each fraction: germinal center B, B220^+^ CD38^−^; transitional B, B220^+^ IgM^hi^ IgD^lo^ CD21^lo^; naive B, B220^+^ IgD^hi^ IgM^lo^; naive T, B220^−^ CD3^+^ CD4^+^ CD62L^hi^ CD44^lo^ LacZ^−^; EM T (LacZ^−^), B220^−^ CD3^+^ CD4^+^ CD62L^lo^ CD44^hi^ LacZ^−^; and EM T (LacZ^+^), B220^−^ CD3^+^ CD4^+^ CD62L^lo^ CD44^hi^ LacZ^+^. (D) Human CD2 and LacZ expression in the peripheral CD4^+^ T-cell fraction in 33-week-old Aicda-cre/R26R/μMT mice. EM T cells (B220^−^ CD3^+^ CD4^+^ CD62L^lo^) in the spleen (SPL) or lymph node (LN) were examined for each mouse with the indicated genotype. Percentage within the gate is indicated.

AID expression in GC B cells is associated with the environment, in which T-B interaction plays a critical role. To examine the possibility that the *Aicda* expression in T cells depends on B cells, we crossed the Aicda-cre/R26R mouse with the μMT mouse, which has virtually no B cells (Figure 4A) [34], [35]. As shown in Figure 4B, there was no difference in the percentage of exAID cells found among the EM T cells in the μMT versus wild-type (WT) background. We conclude that T-B interaction is not essential for exAID T-cell generation, and that exAID T cells can develop without the GC reaction.

### Endogenous AID mRNA expression in T cells

To examine whether the endogenous *Aicda* gene is expressed in T cells, we performed RT-PCR on LacZ^+^ and LacZ^−^ EM T cells, as well as naive T cells, sorted from pooled spleen and lymph node cells. A TaqMan probe that detects the exon 1–2 region of AID mRNA was used, because it does not detect the Aicda-cre transgene product. We detected weak but clear signals from the LacZ^+^ CD4^+^ T cells (Figure 4C). The signal strength was one-tenth of that from immature B cells, and similar to that of mature B cells. Because Cμ and CD19 mRNAs were almost absent in each sample, B-cell contamination was unlikely. Furthermore, we did the same experiment with Aicda-cre/R26R/μMT mice, and detected substantial AID mRNA levels in the LacZ^+^ T cells. These levels were higher than in WT LacZ^+^ T cells, and as high as in immature B cells (Figure 4C). In accordance with the PCR results, the hCD2 levels were much higher in the lymph node T cells of Aicda-cre/R26R/μMT mice than in those of WT mice (Figure 4D). We conclude that LacZ^+^ CD4^+^ T cells indeed expressed endogenous AID mRNA.

### Properties of exAID T cells

**Figure 5:**
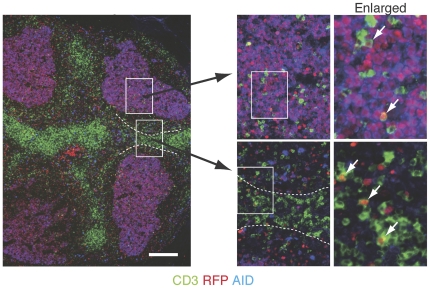
The localization of exAID CD3^+^ cells in Peyer's patches. Left panel: an image of a Peyer's patch, taken with a low power field, stained with CD3 (green), AID (blue), and RFP (red). The section was cut at the position where the germinal center was dense. The dotted line indicates part of a T-B border. Bar represents 200 µm. Right panels: enlarged view of the area indicated in the left panel. White arrows indicate CD3^+^ RFP^+^ double-positive cells.

In T cells, *Aicda* expression occurred primarily in the activated CD4^+^ fraction. To examine whether there are functional differences between exAID and non-exAID cells, we first analyzed the localization of exAID T cells within lymphoid organs. Sections of a Peyer's patch from an Aicda-cre/Rosa-tdRFP mouse were stained with antibodies for CD3 and AID. A cluster of RFP^+^AID^+^ cells, which indicated GCs, included dispersed CD3^+^ cells (Figure 5). Scattered RFP^+^AID^+^ B cells could be seen at the edge of the T-B zone, which was consistent with our previous report (Figure 5) [36]. CD3^+^RFP^+^ T cells were seen throughout the area, including the GC, T zone, and T-B border (Figure 5). These results suggest that the exAID T cells may not have a special localization within lymphoid tissue.

**Figure 6:**
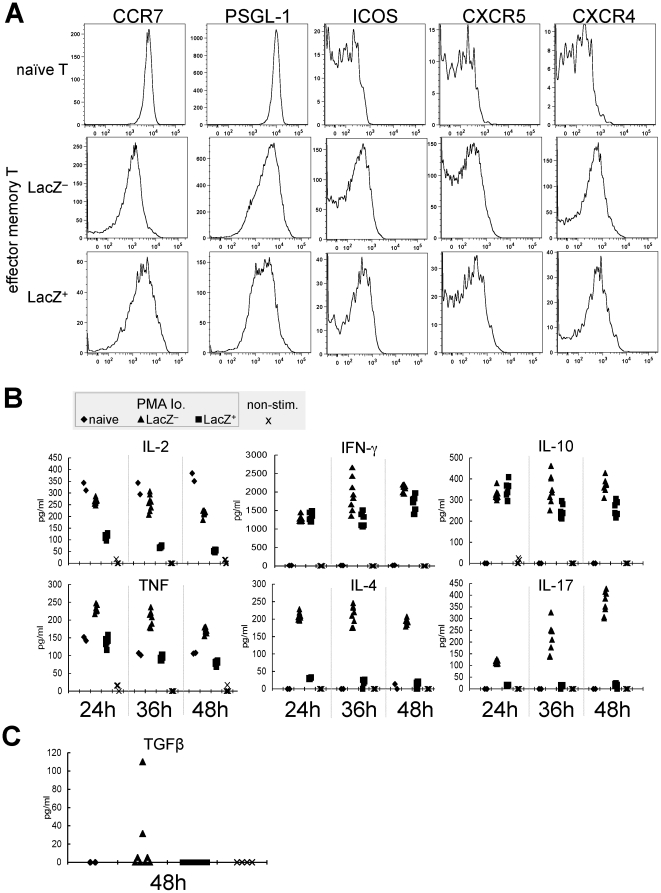
Distinct functional feature of exAID T cells. (A) Naive T (CD3^+^ CD4^+^ CD62L^hi^ CD44^lo^) and EM T (CD3^+^ CD4^+^ CD62L^lo^ CD44^hi^) cells, with or without LacZ, from Aicda-cre/R26R spleens were gated and examined for the indicated molecules. (B) Cytokine production of naive (diamond) and EM (LacZ^−^, triangle; LacZ^+^, square) T-cell fractions after stimulation with PMA and ionomycin is shown for the indicated time points. Unstimulated LacZ^−^ EM cells (non-stim., cross) were used as negative controls. Each symbol represents the result of an individual sample of multiple cultures (naive, n = 2; LacZ^+^, n = 6; LacZ^−^, n = 8; non-stim., n = 4). (C) TGF-β production 48 hours after PMA ionomycin addition, measured by ELISA. Symbols are the same as in (B). The result is representative of three independent experiments. IL, interleukin; IFN, interferon; TNF, tumor necrosis factor.

Consistent with the histological observations, there was no difference in the expression of CXCR4 and CXCR5, which determine cellular localization in the lymphoid organ, between exAID and non-exAID EM CD4^+^ T cells (Figure 6A). However, we observed small but recognizable differences in the CCR7 and PSGL-1 levels (Figure 6A), suggesting that the exAID cells might have acquired some type of functional difference. On the other hand, there was no difference in ICOS, an important co-stimulatory molecule for T-B interaction (Figure 6A).

Older animals bear a unique T-cell subset with diminished reactivity, namely PD-1^+^ memory phenotype CD4^+^ T cells [37]. Because the fraction of exAID T cells increased with age, we checked for PD-1 in LacZ^+^ T cells from the spleen of aged Aicda-cre/R26R mice. The majority of CD62L^lo^LacZ^+^CD4^+^ T cells expressed PD-1, while PD-1 was barely detectable in the CD62L^hi^ naïve T cells (Figure S3D). The CD62L^lo^ LacZ^−^ CD4^+^ T cells were a mixture of PD-1-positive and -negative cells (Figure S3D). Therefore, the exAID T cells found in aged mice seem to be included in a population of PD-1^+^ memory CD4^+^ T cells that has been previously reported [37].

CD4^+^ T cells can be classified into several subsets, such as Th1, Th2, and Th17, according to their cytokine profiles [38]–[40]. Each subset can be induced by different sets of stimuli [39], [40]. If the *Aicda* expression in CD4^+^ T cells is associated with specific activating conditions, the cytokine profile of the exAID subset could be biased. To examine the cytokine signature, we purified LacZ^+^ EM CD4^+^ T cells and other control cells, and then stimulated them by phorbol myristate acetate (PMA) and ionomycin. Naive cells secreted interleukin (IL)-2 and TNF-α, but no other cytokines typical to Th1, Th2, or Th17 (Figure 6B). The LacZ^−^ EM T cells secreted all of the cytokines examined, indicating that, as expected, this population contained various types of Th-cell subsets. By contrast, the exAID T cells produced lower levels of IL-2 and negligible amounts of IL-4, IL-17, and TGF-β. However, the IL-10 and interferon-γ productions were comparable to those of LacZ^−^ EM T cells (Figure 6B and 6C). The exAID T cells also produced TNF-α at a level similar to the naive T cells. Stimulation with anti-CD3 plus anti-CD28 gave essentially the same results (Figure S4A). The bias in the cytokine signature suggests that *Aicda* expression may be associated with specific signals.

To examine whether *in vitro* stimulation can induce *Aicda* expression in T cells, we cultured naive CD4^+^ T cells with combinations of various cytokines that can induce either Th1, Th2, or Th17 cells *in vitro*. However, none of these culture conditions induced LacZ^+^ T cells (Figure S4B). Given the slow accumulation of LacZ^+^ T cells *in vivo* (Figure 3D), the frequency of *Aicda* expression in T cells should be so low that it would not be detectable in short-term culture. Alternatively, *Aicda* expression might require a unique *in vivo* environment that is absent in the culture system.

**Figure 7:**
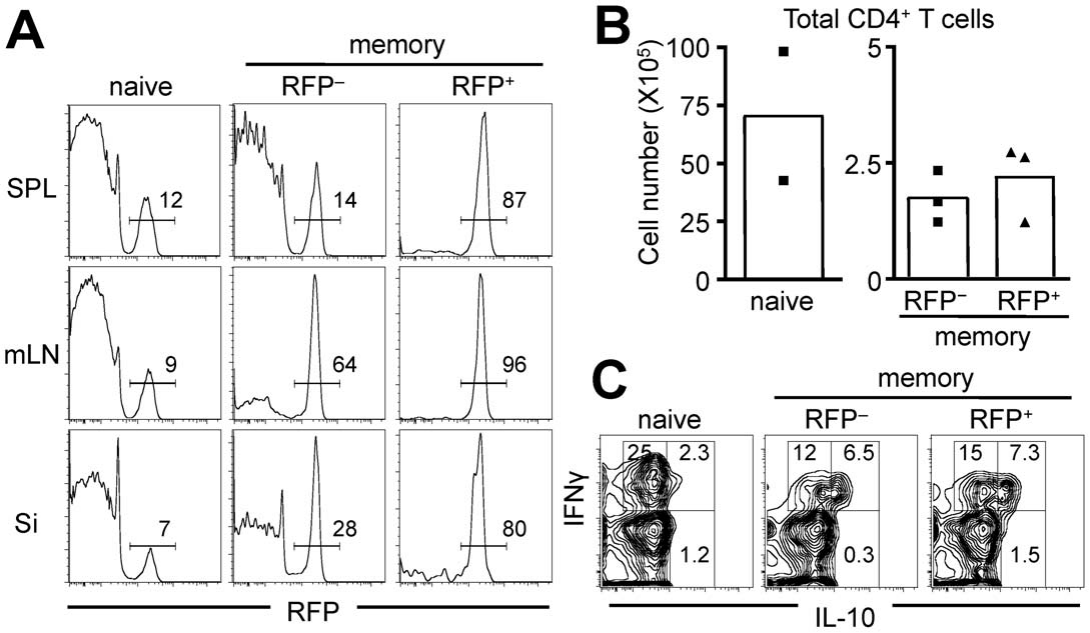
Generation of exAID CD4^+^ T cells *in vivo* by Adoptive transfer to CD3ε^−/−^ mice. (A) RFP expression of CD4^+^ T cells recovered from spleen, mesenteric lymph node, and small intestine of CD3ε^−/−^ mice transferred indicated phenotype of T cells. (B) CD4^+^ T cells that were recovered from the spleen, mesenteric lymph node, Peyer's patch, small intestine and large intestine of the mice transferred RFP^−^ EM (n = 3; square) and RFP^+^ EM CD4^+^ T cells (n = 3; triangle) as well as naïve T cells (n = 2; square) were pooled; the total number of cells was counted and plotted. Average numbers are shown by column graphs. (C) IFN-γ and IL-10 double producing cell from EM CD4^+^ T cells after the adoptive transfer. IFN-γ and IL-10 expression in spleen CD4^+^ T cells obtained from T-cell-transferred CD3ε^−/−^ mice were analyzed by intra-cellular cytokine staining. Recovered cells were stimulated with PMA and ionomycin for 4 hours before the staining. SPL, spleen; mLN, mesenteric lymph node; Si, small intestine; memory, effector memory.

In contrast, adoptive *in vivo* transfer of naïve CD4^+^ T cells or Aicda-cre/RFP^−^ EM CD4^+^ T cells into T cell-deficient (CD3ε^−/−^) mice revealed that a considerable fraction of cells expressed *Aicda* and become RFP^+^ cells (Figure 7A). The frequency of RFP^+^ T cells generated from the RFP^−^ EM T cells was much higher than that observed from naïve T cells. Furthermore, the RFP^+^ cells generated from RFP^−^ EM T cells appeared to be located predominantly in the mesenteric lymph nodes (mLN) and small intestine (Si) lamina propria while majority remained RFP^−^ in the spleen, suggesting that RFP^+^ EM T cells might be generated upon activation of EM T cells by environmental stimuli in the gut (Figure 7A). Both RFP^+^ and RFP^−^ EM phenotype T cells did not expand in CD3ε^−/−^ mice as compared with naïve T cells (Figure 7B). We did not find any difference in the recovered-cell number between the transfer of RFP^+^- and RFP^−^-cells. It suggested that RFP^+^- and RFP^−^-cells have similar capability for proliferation even after the transfer; the result is consistent with the result obtained by cell cycle indicator mice (Figure 7B and Figure 3E). We next explored the cytokine signature of transferred T cells recovered from CD3ε^−/−^ mice. A similar fraction of RFP^+^ and RFP^−^ EM cells became IFN-γ/IL-10 double-producing cells, consistent with their initial cytokine profile before transfer (Figure 7C and Figure 6B). Taken together, *Aicda* expression in T cells seems to be induced under specific *in vivo* conditions in response to stimuli derived from gut environment, with the resultant exAID T cells acquiring distinct functional properties, at least in the cytokine profile.

It is important to know whether the AID in T cells plays any role in T-cell functions. Aberrant AID expression could cause mutations in proto-oncogenes, such as *Myc* [6], [8], [14]). However, we could not find AID-dependent mutations in exAID T cells at the *Myc* mutation hotspot through sequencing (Table 2 and S1). T cell antigen receptor (TCR) genes are other possible SHM targets [14]. We sorted the hCD2^+^ LacZ^+^ T cells from μMT background mice, and a mixture of TCR cDNAs were randomly cloned for sequencing analysis; however, no significant difference in mutation frequency was observed between the LacZ^+^hCD2^+^ and LacZ^−^hCD2^−^ fractions (Table S2). Nevertheless, we cannot exclude the possibility that a limited number of TCR clones accumulated mutations, because the TCR sequencing analysis showed only the average in the total population analyzed.

**Table 2:** Mutation frequency of *Myc* exon 1-intron 1 region.

Exp.	Cell	Mutation rate(/base pair)	Mutated base/Total base
Non imm.	Naïve T	0.20×10^−4^	3/153,114
	EM T Rosa^+^	0.40×10^−4^	7/174,408
	EM T Rosa^−^	0.56×10^−4^	2/35,490
Immunized*			
Exp. 1	Naïve T	0.16×10^−4^	3/182,520
	EM T Rosa^+^	0.52×10^−4^	8/153,114
	EM T Rosa^−^	0.34×10^−4^	4/118,235
Exp. 2	Naïve T	<0.14×10^−4^	0/73,340
	EM T Rosa^+^	0.26×10^−4^	3/114,835
	EM T Rosa^−^	0.35×10^−4^	5/143,785

*Mice immunized TNP-ova emulsified with incomplete Freund's adjuvant. EM, effector memory; Exp, experiment; Rosa, LacZ or RFP.

### AID expression in other tissues

AID expression in non-lymphocytes has been observed with viral or bacterial infection, or under other inflammatory conditions [10], [17]. AID has also been found in non-lymphoid tissues, mouse ovary [25], and human testis [26] under normal conditions. We therefore histologically examined non-lymphoid tissues, including the testis and ovary of Aicda-cre/Rosa-tdRFP mice, and found RFP-positive cells in both the seminiferous tube and the ovary (Figure S5AB). However, only one or two tubes per tissue section contained RFP^+^, suggesting that *Aicda* is occasionally expressed in a few spermatocytes in normal spermatogenesis. In the ovary, many granulosa cells and Theca externa cells were RFP-positive. We could not find RFP^+^ oocytes so far as we examined. We also could not find clear RFP signals in any other tissues examined so far, which include the pancreas, cerebellum, cerebrum, lung, liver, and kidney (unpublished observations).

## Discussion

In the present study, we employed a highly sensitive monitoring system for *in vivo Aicda* promoter activation and found a new fraction of CD4^+^ T cells that expressed *Aicda* at some point. This observation convincingly demonstrates that AID can be expressed in non-B somatic cells under physiological conditions. Such T-cell populations may not be discovered by conventional procedures, because less than 1% of the CD4^+^ T cells expressed hCD2 or AID at any given time point. Despite this low frequency, exAID CD4^+^ T cells accumulated with age, eventually making up about 25% of the CD4^+^ T-cell population. This raises the possibility that these cells play distinctive functions in long-term phenomena such as immune-memory or immunosenescence, rather than in short-term responses.

Kwon et al. (2008) demonstrated that the efficiency of Cre-mediated deletion induced by the Aicda-cre transgene varies between the different floxed reporters present in the *Tcfe2a^floxed^* and *R26R^eyfp^* alleles. Only 20% of the GC B cells recombine *R26R^eyfp^*, whereas almost all of the GC B cells recombine *Tcfe2a^floxed^*, suggesting that Cre's accessibility to a given locus is an important factor for conditional mutagenesis. Although the two reporter genes used in this study were inserted into the *Rosa26* locus, which is similar to the *R26R^eyfp^* allele, both of them allowed us to detect Cre activity with apparently higher sensitivity than that obtained with the *R26R^eyfp^*. Therefore, the sensitivity of the *Rosa26* system also seems to depend on the structure of the integrated reporter gene itself. Furthermore, despite its high efficiency in GC B cells, the *Tcfe2a^floxed^* allele fails to reveal Aicda-cre activity in immature B and T cells [27], suggesting that Cre's accessibility to a given target gene may also vary in different cell types and developmental stages. These results imply that the fluctuating factors discussed above strongly influence the outcome of Cre-mediated conditional mutagenesis. Importantly, however, the Cre-positive cells detected with the more sensitive reporter system used in this study could not have been false positives, because they both depended on Aicda-cre and expressed *Aicda* transcripts, as shown by RT-PCR.

The exAID T cells expressed IFN-γ and IL-10, but little IL-4 or IL-17, indicating that they do not match any typical CD4^+^ T-cell subset. Actually, substantial fraction of cells expressed both IFN-γ and IL-10 as obvious in the intracellular staining. The IFN-γ production implies Th1, but IL-10, which has broad anti-inflammatory activity, has not been considered a typical Th1 cytokine [41]. Recently, however, cells producing IL-10 have been found in chronic infections caused by protozoa such as *Toxoplasma gondii* and *Leishmania major* [42], [43]. These IL-10-producing Th1 cells are the major source of IL-10 in mice suffering from chronic intracellular parasite infection. IL-10 protects host animals from mortal hyperinflammation by *T. gondii*, while it prevents the healing of skin lesions in animals infected with *L. major* [42], [43]. Although our mice were not infected with such protozoa, environmental antigens might occasionally stimulate T cells, thereby activating the regulatory cytokine program. Besides, the exAID EM T cells express slightly higher CCR7 and lower PSGL-1 in comparison with the non-exAID EM T cells. Because CCR7 and PSGL-1 is involved in leukocyte homing to lymphoid organ and effector site, respectively, exAID T cells might have some tendency closer to the central memory T cells although CD62L expression were not high [32]. The fact that exAID T cells acquired a cytokine signature and a homing receptor pattern distinct from other T cells suggests that the *Aicda* expression in T cells is associated with some specific stimuli.

A recent report demonstrated a unique PD-1^+^ T-cell subpopulation that is characteristic of aged animals [37]. These cells have memory phenotypes but hardly proliferate in response to TCR stimulation, and they produce fewer typical T-cell cytokines [37]. We found that most of the exAID CD4^+^ T cells in the spleen of aged animals were PD-1^+^, but many PD-1^+^ cells remained LacZ-negative. Therefore, it is possible that a fraction of the PD-1^+^ memory phenotype CD4^+^ T cells might comprise of heterogeneous cells, some of which express *Aicda* sometime during their development. Interestingly, C/EBPα, which has a binding motif involved in *Aicda* expression [18], is highly expressed in the PD-1^+^ T-cell population [37].

What kind of stimuli might be involved in *Aicda* expression in T cells? *Aicda* expression was not induced in T cells in our *in vitro* culture experiments with various polarizing conditions; thus the conventional stimulation to activate T cells is not sufficient to induce *Aicda* expression efficiently. In contrast, a considerable fraction of effector memory cells as well as naïve CD4^+^ T cells became RFP^+^ upon *in vivo* transfer into T cell-deficient mice, suggesting that AID upregulation may take place during homeostatic expansion, induced likely by antigens from the commensal bacteria [44], [45]. It has been reported that IL-10-producing Th1 cells are generated by stimulation with a high antigen dose in conjunction with IL-12 *in vitro* [46]. Therefore, strong antigen stimulation might also be involved in generating exAID T cells. TCR crosslinking induces NF-κB signaling [47], [48], which can also promote *Aicda* expression [18]. NF-κB can also be activated through Toll-like receptors, which are important for *Aicda* expression in immature B cells [20]. Th1 cells express TLR2 [49], which binds to bacterial components and activates NF-κB. Our finding that AID was more prominently expressed in the μMT background may reflect the increased environmental stimuli under such an immunodeficient condition. That is, in the absence of normal Ig production, the homeostasis of intestinal microbiota could be severely disturbed; causing increased systemic stimulation by aberrant bacterial components [50] and increasing the chance of NF-κB activation in T cells.

The biological significance of AID expressed in T cells is unclear. Although SHM on *Tcr* has been reported, we were not able to detect it [51]; however, we cannot exclude the possibility that a limited number of T-cell clones accumulated TCR mutations. On the other hand, because the *Aicda* expression in T cells is not abundant, we cannot exclude the possibility that the expression simply results from a leaky transcriptional activation and has no functional relevance. Another aspect of AID expression is its involvement in tumorigenesis, since AID overexpression *in vivo* efficiently generates T-cell tumors [14]. Although we could not detect significant AID-induced *Myc* mutations in the exAID T cells, it is possible that even rare AID expression can still increase the probability of tumor formation. In this context, it is noteworthy that human T cell leukemia virus 1 (HTLV-1) infection, which causes T cell leukemia after a prolonged course, can induce *Aicda* expression in infected T cells [52], [53].

Immature B cells have been reported to express low levels of AID, for which the Toll-like receptor signal is responsible [20]. SHM in immature B cells has been proposed to contribute diversity to the primary repertoire [20]–[22], [54]. It is not clear, however, whether such mechanisms are critical for B-cell repertoire maintenance. ExAID cells comprised only 5–10% of immature B cells, and the average level of AID mRNA expression was less than 10% of that in GC B cells. *Aicda* expression in immature B cells may be induced or triggered in part of the population, rather than intrinsically programmed. The AID expressed in immature B cells has been suggested to play a role in the negative selection of auto-reactive B cells [20], [23], [24]. ExAID immature B cells might have expressed BCR reactive to environmental antigens, including self-antigens, and such BCR could be edited to change its specificity.

Recently, two groups reported analogous strategies for analyzing AID expression in memory B cells [55], [56]; these reports did not study AID in immature B cells, as our study did. Differences may be due at least partly to the *cre* expression level or the reporter sensitivity. Dogan et al. used the cre-ER system, in which cre is inactive until tamoxifen is administered [56]. This system is excellent for labeling the cells at one particular moment by tamoxifen injection, but it does not allow the accumulation of labeled exAID cells. Despite these differences, all the systems clearly detected AID-expressing B cells in the GC. ExAID B cells, which have been proposed to be memory B cells [56], actually accumulated after 40 weeks of age. However, the majority of exAID cells were non-switched cells (IgM^+^ IgD^+^), even in very old animals. This may suggest that switched memory B cells are not a major population. Alternatively, such cells may emigrate from the spleen. The importance of memory plasma cells in humoral memory has recently been reinforced [57]. It is possible that switched cells preferentially differentiate to plasma cells, as earlier proposed [56].

Several groups have reported the presence of AID mRNA in germ cells [25], [26], [58], [59]. It has been proposed that AID plays a critical role in reprogramming the DNA epigenetic mark. However, it is difficult to reconcile this with the long-term observation that AID knockout mice are fertile and physiologically normal. We only occasionally detected an RFP-positive seminiferous tubule; therefore, the expression is unlikely to be a developmentally programmed event that occurs in every cell. In the ovary, consistent with previous reports, we observed many RFP^+^ granular and Theca cells in ovarian follicles, whereas the oocytes appeared to be RFP^−^. We cannot exclude the possibility that our detection system is limited. Moreover, in contrast to sperm, the number of oocytes that could be examined by our procedure is limited. Although we do not exclude the possibility that some of oocytes occasionally express the *Aicda* gene, it is clear that AID expression in non-lymphoid tissues is either very low or a rare event under normal conditions. Therefore, strong pathogen stimulation is likely to be needed to trigger AID expression in non-lymphoid cells, which could lead to tumorigenesis.

## Materials and Methods

### Mice

Aicda-cre [27], R26R [29], Rosa-tdRFP [28], Fucci [33], μMT [34] and CD3ε^−/−^ [60] mice were bred and maintained in specific pathogen-free conditions at the Institute of Laboratory Animals, Kyoto University, and in the animal facility at RIKEN. These animals were backcrossed at least 7 times on a C57BL/6 background. All experimental protocols involving animal use were approved by the Kyoto University Animal Research Committee and/or the Institutional Animal Care and Use Committee at RIKEN (Protocol number MedKyo10055).

### Antibody staining, LacZ detection and cell sorting

Cells were suspended in FACS buffer (PBS supplemented with 4% FBS, 1 mM HEPES, and 0.6% sodium citrate). The following antibodies were used for staining: monoclonal antibodies conjugated with FITC, PE, PE-Cy7, APC, APC-Cy7, Biotin or Pacific-Blue, specific for B220 (RA3-6B2), CD19 (1D3), CD93 (AA4.1), IgM (R6-60.2), IgD (11-26), CD38 (90), CD3 (500A2), CD4 (GK1.5), CD8 (53-6.7), CD44 (IM7), CD62L (MEL-14), NKG2D (CX5), Dx5, NK1.1 (PK136), CCR7 (4B12), PSGL-1 (2PH1), ICOS (15F9), CXCR5 (2G8), CXCR4 (2B11), or hCD2 (RPA-2.10) (BD Biosciences or eBioscience). FITC conjugated or biotinylated PNA (VECTOR Laboratories) was also used. Biotinylated reagents were detected with either streptavidin-APC-Cy7 or streptavidin-APC (BD Biosciences). For LacZ detection, equal volumes of cell suspension and 2 mM fluorescein-di-β-galactopyranoside (FDG) in H_2_O were mixed at 37°C for 1 min. Stained cells were analyzed and sorted by FACS CantoII (BD Biosciences) and FACSAria, respectively, and analyzed with FlowJo software (TreeStar, Inc.). A live lymphocyte population was gated according to forward-side scatters and 7-amino-actinomycin D staining.

### Cell culture and cytokine detection

Sorted cells were cultured with PMA (50 ng/ml) and ionomycin (1 µg/ml) in RPMI-1640 supplemented with 10% fetal calf serum, 2 mM L-gln, 100 µg/ml penicillin, 100 µg/ml streptomycin, and 1 mM sodium pyruvate. In some experiments, cells were stimulated by pre-coating plates with 2 µg/ml anti-mouse CD3ε (145-2C11, eBioscience) and 2 µg/ml anti-mouse CD28 (37.51, eBioscience) with or without added cytokine combinations, including TGF-β (3 ng/ml), IL-6 (20 ng/ml), IL-12 (5 ng/ml; BD Pharmingen), or IL-4 (10 ng/ml; R&D Systems). Concentrations of IL-2, IL-4, IL-10, IL-17, IFN-γ, and TNF-α in culture supernatants were measured by ELISA, a Ready-SET-Go kit (eBioscience), a CBA Mouse Th1/Th2/Th17 cytokine kit (BD Bioscience), or a TGF-β1 Emax ImmunoAssay System (Promega), used according to the manufacturer's protocol.

### Quantitative Real-time PCR

Total RNA was extracted by TRIzol reagent (Invitrogen Life Technologies), and cDNA was synthesized with TaqMan® Reverse Transcription Reagents (Applied Biosystems) or a LightCycler RNA Pre-Amplification Kit (Roche). The *Aicda*, *Igh-M*, *Cd1*9, and *Gapdh* expressions were assessed with TaqMan® Gene Expression Assays (Applied Biosystems; Mm00507774_m1, Mm01718956_m1, Mm00515428_m1, and Mm99999915_g1) using Applied Biosystem's 7900HT PCR system. Gene expression was normalized to the *Gapdh* or *Ppib* expression as assessed by iQ Sybr green supermix (Bio-Rad Laboratories, Inc.) with cyclophilin-F and cyclophilin-R primers (Table S3).

### Immunohistochemistry

Histological staining was done essentially as described elsewhere [61]. Briefly, tissues were fixed in 4% paraformaldehyde at 4°C, incubated in 30% sucrose at 4°C overnight, embedded in OCT compound (Sakura Finetechnical), snap-frozen in liquid N_2_, and then sectioned at a thickness of 8 µm with a Cryostat. The sections were blocked in TNB buffer (PerkinElmer Life Science) containing 5% normal donkey serum, using the Streptavidin/Biotin Blocking Kit (Vector Laboratories). Endogenous peroxidase activity was quenched with 1% H_2_O_2_. The primary antibodies were applied in TNB buffer for 1.5 h at room temperature, or overnight at 4°C. Slides were washed and incubated with biotin-conjugated secondary antibodies, and then incubated with streptavidin–HRP conjugate (Zymed Laboratories). Antigens were detected using tyramide-FITC or tyramidetetramethylrhodamine from the tyramide signal amplification kit (PerkinElmer Life Science) according to the manufacturer's instructions. Stained slides were mounted with Fluoromount-G (Southern Biotechnology Associates). To observe RFP without immunostaining, slides were prepared as above, and then mounted with Slow fade Gold with DAPI (Invitrogen). Antibody used for AID staining was MAID-2 [62].

### Sequencing for *Ig*, *Myc* and *TCRs*

The *IgV* downstream region was amplified and sequenced as described [4]. The 1-kb region of Myc exon1-intron1 was amplified by PCR from the genomic DNA of naïve, LacZ^+^ EM, and LacZ^−^ EM T cells with the high fidelity polymerase Pyrobest (TaKaRa), using Myc-F and Myc-R primers for 35 cycles. PCR products were first A-tailed by Taq, and then cloned by the T-vector pMD20 (TaKaRa) for sequencing. In some experiments, mice were immunized once with 100 µg of trinitrophenyl (TNP)-ova in complete Freund's adjuvant, and boosted 4 times per 2-week interval with 50 µg TNP-ova in incomplete Freund's adjuvant.

RNA from sorted T cells was reverse-transcribed with a mixture of specific primers for TCR Cα and Cβ (Cα-1 and Cβ-1). Synthesized single-stranded cDNA was purified with the Affymetrix cDNA clean-up module (Affymetrix). Single-stranded DNA was tailed by terminal deoxynucleotide transferase (New England Biolabs) with 190 µM dGTP for 10 minutes at 37°C, purified with the Affymetrix cDNA cleanup module, and then used for PCR with a mixture of primers (Cα-2-Xho, 0.3 µM; Cβ-2-Xho, 0.3 µM; anchor-Xba, 0.3 µM; anchor+dC-Xba, 0.05 µM) by Prime STAR HS (TaKaRa) for 35 cycles. PCR products around 400–800 bp in size were purified by agarose gel electrophoresis, then randomly cloned into a pBluescript vector at XbaI and XhoI sites for sequencing. Sequence data was searched on IMGT/GENE-DB [63] and on NCBI blast.

### Adoptive transfer

Spleen and lymph node cells of Aicda-cre/Rosa-tdRFP mice were pooled and used for the sorting. One hundred thousand of CD62L^hi^CD44^lo^RFP^−^ naïve T cells, 1×10^5^ CD62L^lo^CD44^hi^RFP^−^ and 1×10^5^ CD62L^lo^CD44^hi^RFP^+^ EM T cells were injected intravenously into CD3ε^−/−^ mice. They were analyzed at 10 weeks after the transfer.

### Statistics

P values (two-tailed) were calculated by Fisher's exact test. Correlation coefficient between age and percentage of exAID lymphocytes were calculated by Excel software (Microsoft)

## Supporting Information

Figure S1 **Characterization of Aicda-cre mouse crossed with Rosa reporter mice.** (A) Schematic representation of Aicda-cre, R26R and Rosa-tdRFP alleles. (B) FACS analysis of B220^+^ cells for hCD2 and RFP expression in peripheral lymphoid tissues of Aicda-cre/Rosa-tdRFP mice (31-week) are shown. CD95 (Fas) is an activated B cell marker. (C) Estimated absolute numbers of Rosa marker (R26R, diamond; Rosa-tdRFP, cross) positive hCD2^−^ B220^+^ exAID B cells were plotted by age. (D) Ig isotype of LacZ^−^ and LacZ^+^ B cells in the spleen of aged mouse (64-week). The result is representative for three independent staining experiments. SPL, spleen; LN, lymph node; PP, Peyer's patch.
(TIFF)

Figure S2 **ExAID CD4^+^ T cells visualized by Rosa-tdRFP system.** (A) (B) Spleen cells from Aicda-cre/Rosa-tdRFP mouse (33-week, upper panels) were stained with indicated markers. RFP positive CD4^+^ T cells appeared Aicda-cre dependent manner. (B) RFP histograms of the CD3^+^CD4^+^ fraction were shown. (C) Spleen (SPL), lymph node (LN) and Peyer's patch (PP) T cells were immuno-stained and gated as indicated.
(TIFF)

Figure S3 **Analysis of non-B exAID cells.** (A) NK cells contain a small fraction of cells with *Aicda* expression history. Spleen cells from Aicda-cre/R26R mouse (21-week) were stained by NK cell markers. Almost no NK marker positive cells are detected within CD3^+^ CD4^+^ T cells while CD3^−^ CD4^−^ contains few but obvious LacZ^+^ NK marker positive cells. The number indicates percent of the fraction in each quadrant among gated cells. The result was representative for three independent experiments with 1 or 2 mice. (B) Estimated absolute numbers of Rosa marker (R26R, diamond; Rosa-tdRFP, cross) positive CD4^+^ B220^−^ exAID T cells were plotted by age. (C) Percentage of Rosa marker positive cells (R26R, diamond; Rosa-tdRFP, cross) in cytotoxic T cells fraction in the spleen. The fractions were defined as CD8^+^ (filled diamond) or CD4^−^ (open diamond) T cells. The x-axis is the age of mouse (week). (D) PD-1 expression of naïve (CD62L^hi^), non-exAID effector memory (CD62L^lo^ LacZ^−^) and exAID effector memory (CD62L^lo^ LacZ^+^) helper T cells (CD3^+^ CD4^+^) in aged (65-week) mouse. The result was representative for the analysis of three old mice (65- or 64-week).
(TIFF)

Figure S4 ***In vitro*** **stimulation of CD4^+^ T cells.** (A) CD4 T cells were fractionated to Naïve, and LacZ^+^ and LacZ^−^ effector memory T cells by sorting according to their expression of CD44 and CD62L. They were cultured with 2 µg/ml anti-mouse CD3e (clone 145-2C11, eBioscience) and 2 ug/ml anti-mouse CD28 (clone 37.51, eBioscience) for 2 Days. IL-2, IL-4, IL-17, IFN-γ and TNF-α in culture supernatants were measured by ELISA, Ready-SET-Go kit (eBioscience). The experiment was done twice and the results were essentially the same. (B) *In vitro* stimulation of Naive CD4^+^ cells failed to induce AID expression. Naive CD4^+^ T cells were sorted from Aicda-cre/R26R mice and cultured *in vitro* with various combination of stimulants with which T cells should polarize to different types of helper T cell states as indicated above each panel. The left most is the culture with un-fractionated CD4 T cells that included LacZ^+^ cells as a control culture. The data is the representative of two independent experiments.
(TIF)

Figure S5 **Histological analyses of testis and ovary.** Frozen section of fixed tissues from Aicda-cre/Rosa-tdRFP and control mice (wt) were examined. Nuclear counter staining of DAPI and bright field image (BF) were shown below and above. (A) Images of testis. Boxed regions were magnified as indicated by arrows. A scale bar is shown in the high power magnification picture of the DAPI staining. Scale bar = 25 µm. (B) Images of ovary. Three different follicles of Aicda-cre/Rosa-tdRFP ovary are shown. Two sets of males and one set of female with the same number of controls were examined.
(TIF)

Table S1 **Mutation frequency of** ***Myc*** **exon 1-intron 1 region.**
(PDF)

Table S2 **Mutation analysis on randomly cloned TCR cDNA.**
(PDF)

Table S3 **Primers used in this study.**
(PDF)
