# Supplementary figures and images for: Activation-Induced Cytidine Deaminase Expression in CD4+ T Cells is Associated with a Unique IL-10-Producing Subset that Increases with Age
Source: PLoS One. 2011 Dec 28;6(12):e29141. doi: 10.1371/journal.pone.0029141 (PMC3247255; doi:10.1371/journal.pone.0029141)

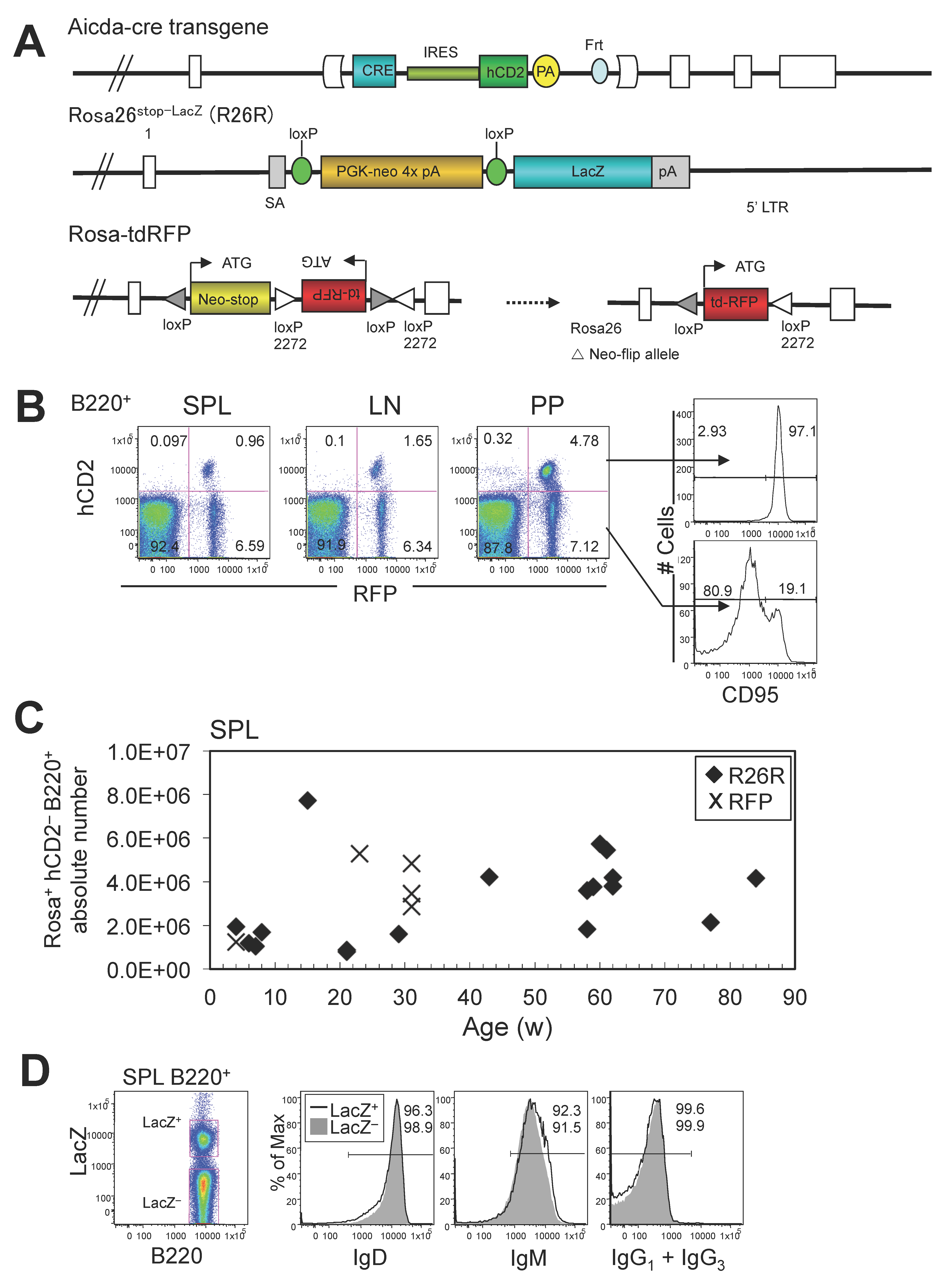

Supplement: Figure S1 — Characterization of Aicda-cre mouse crossed with Rosa reporter mice. (A) Schematic representation of Aicda-cre, R26R and Rosa-tdRFP alleles. (B) FACS analysis of B220+ cells for hCD2 and RFP expression in peripheral lymphoid tissues of Aicda-cre/Rosa-tdRFP mice (31-week) are shown. CD95 (Fas) is an activated B cell marker. (C) Estimated absolute numbers of Rosa marker (R26R, diamond; Rosa-tdRFP, cross) positive hCD2− B220+ exAID B cells were plotted by age. (D) Ig isotype of LacZ− and LacZ+ B cells in the spleen of aged mouse (64-week). The result is representative for three independent staining experiments. SPL, spleen; LN, lymph node; PP, Peyer's patch. (TIFF) [file pone.0029141.s001.tiff]

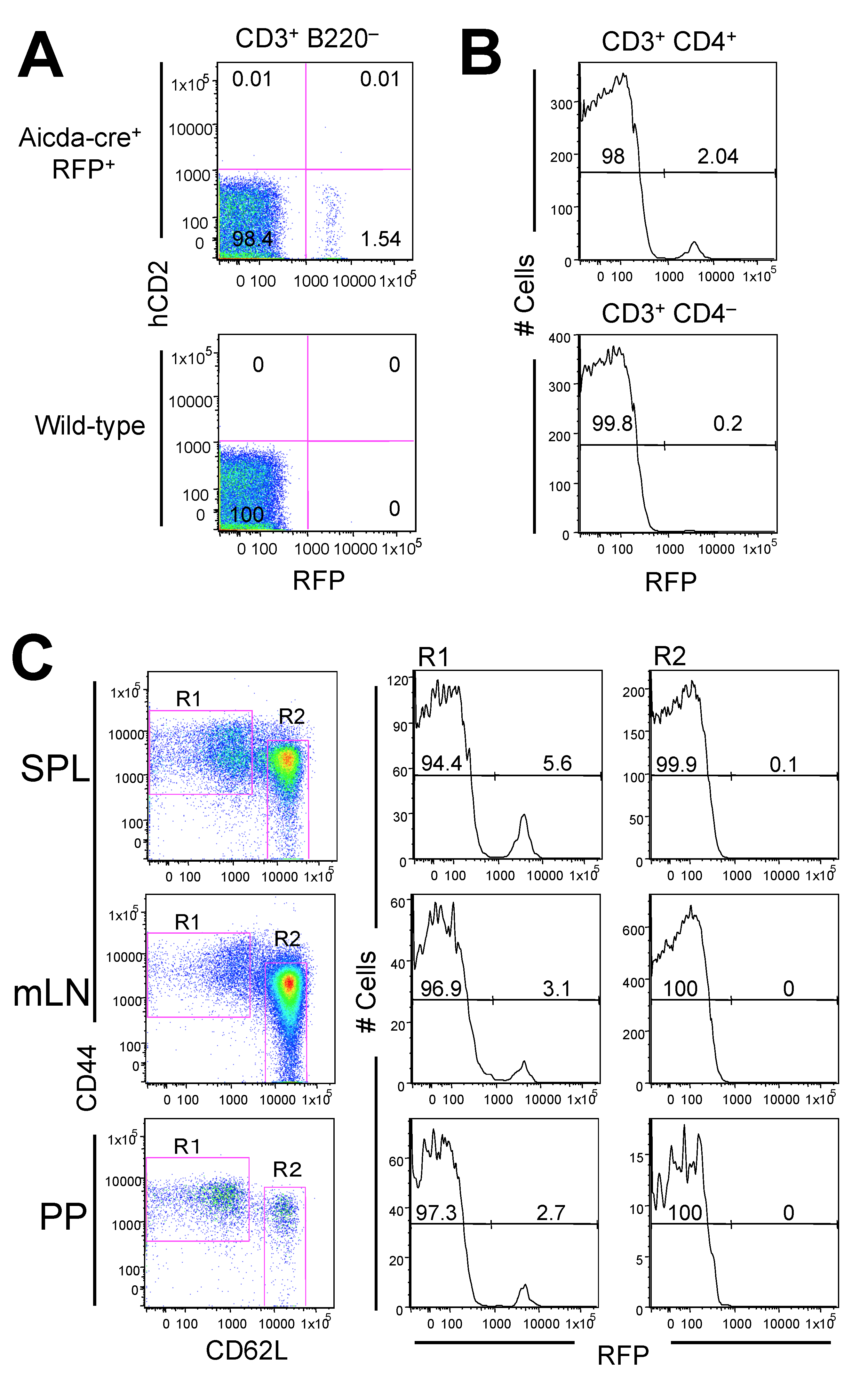

Supplement: Figure S2 — ExAID CD4+ T cells visualized by Rosa-tdRFP system. (A) (B) Spleen cells from Aicda-cre/Rosa-tdRFP mouse (33-week, upper panels) were stained with indicated markers. RFP positive CD4+ T cells appeared Aicda-cre dependent manner. (B) RFP histograms of the CD3+CD4+ fraction were shown. (C) Spleen (SPL), lymph node (LN) and Peyer's patch (PP) T cells were immuno-stained and gated as indicated. (TIFF) [file pone.0029141.s002.tiff]

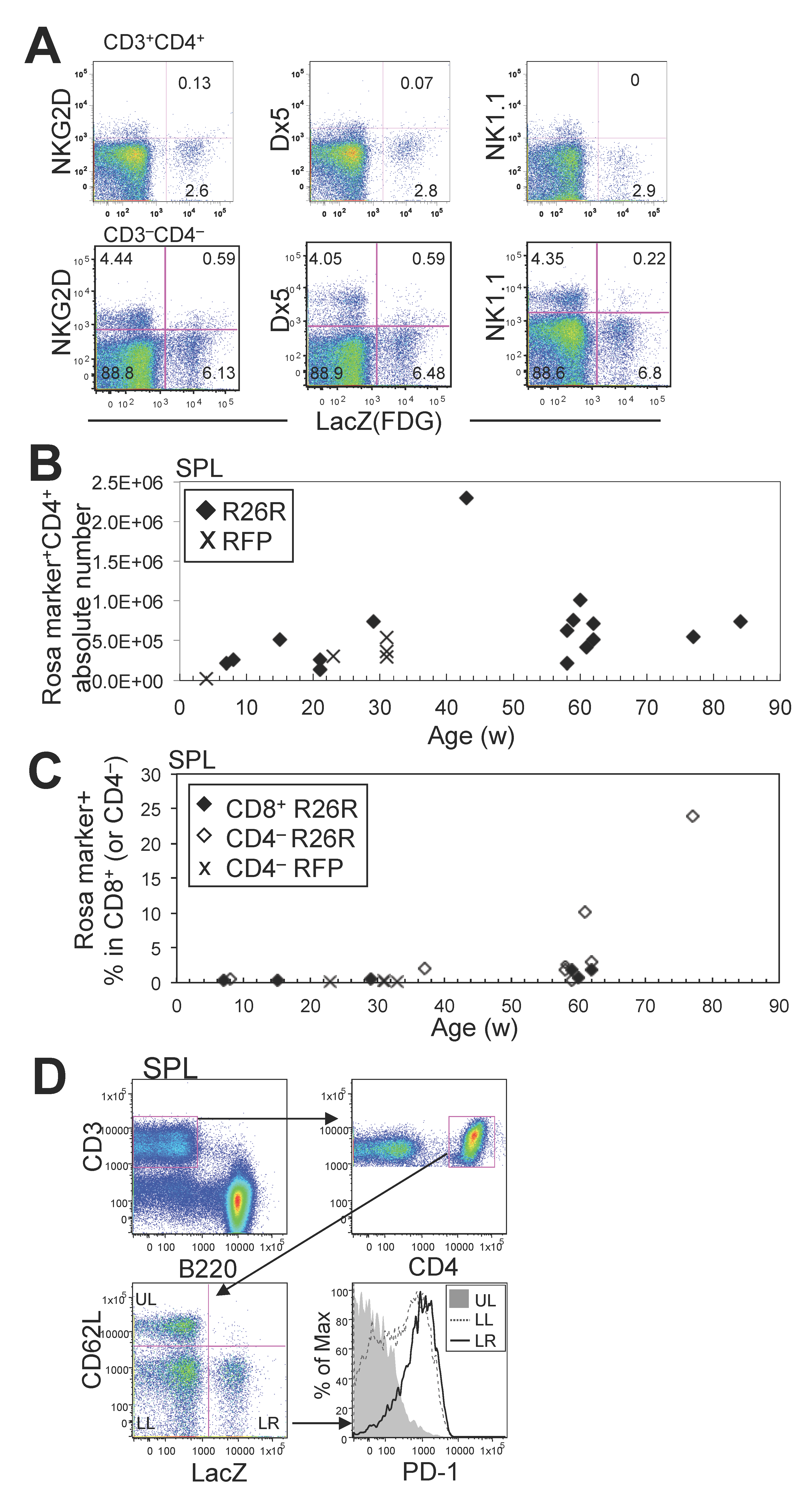

Supplement: Figure S3 — Analysis of non-B exAID cells. (A) NK cells contain a small fraction of cells with Aicda expression history. Spleen cells from Aicda-cre/R26R mouse (21-week) were stained by NK cell markers. Almost no NK marker positive cells are detected within CD3+ CD4+ T cells while CD3− CD4− contains few but obvious LacZ+ NK marker positive cells. The number indicates percent of the fraction in each quadrant among gated cells. The result was representative for three independent experiments with 1 or 2 mice. (B) Estimated absolute numbers of Rosa marker (R26R, diamond; Rosa-tdRFP, cross) positive CD4+ B220− exAID T cells were plotted by age. (C) Percentage of Rosa marker positive cells (R26R, diamond; Rosa-tdRFP, cross) in cytotoxic T cells fraction in the spleen. The fractions were defined as CD8+ (filled diamond) or CD4− (open diamond) T cells. The x-axis is the age of mouse (week). (D) PD-1 expression of naïve (CD62Lhi), non-exAID effector memory (CD62Llo LacZ−) and exAID effector memory (CD62Llo LacZ+) helper T cells (CD3+ CD4+) in aged (65-week) mouse. The result was representative for the analysis of three old mice (65- or 64-week). (TIFF) [file pone.0029141.s003.tiff]

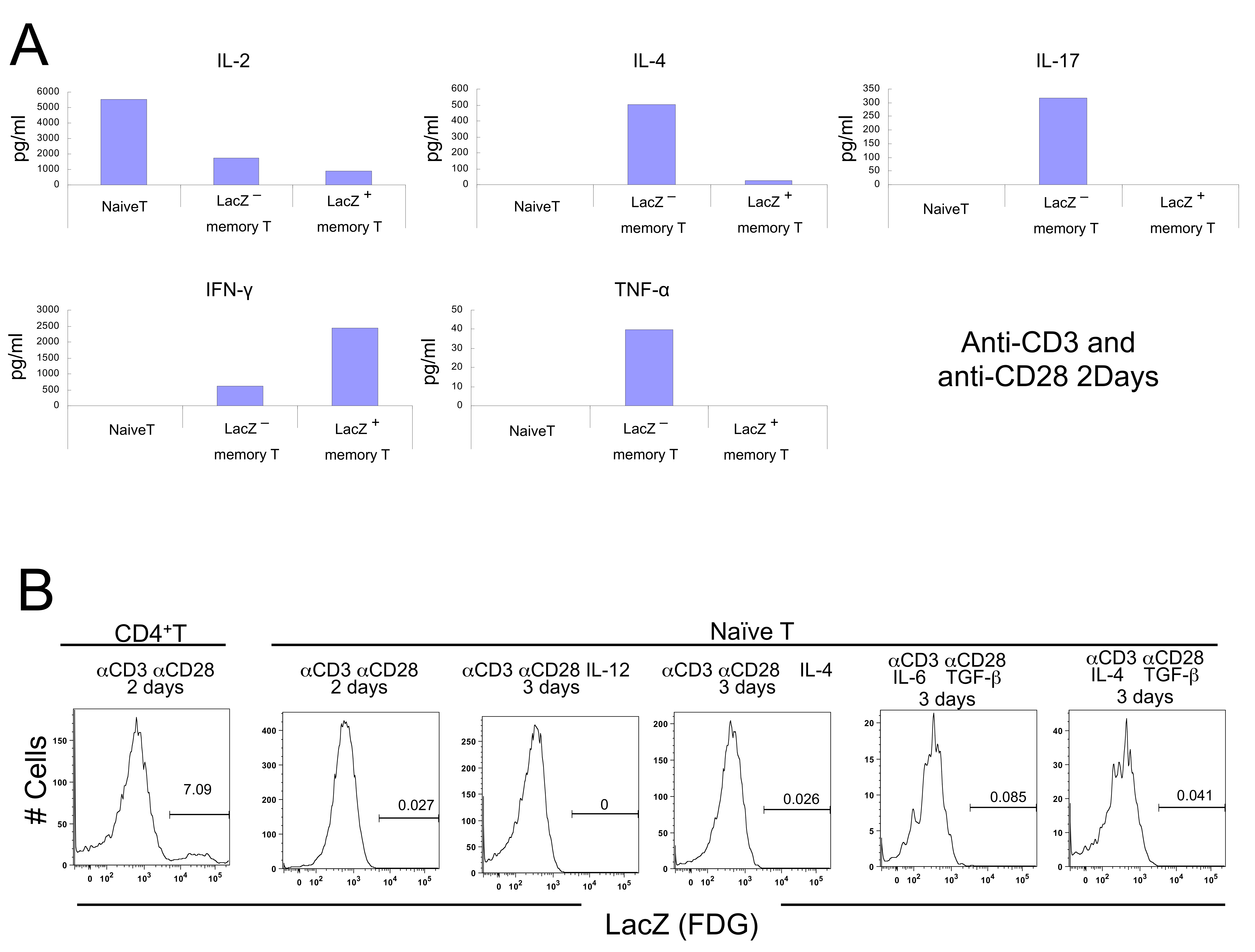

Supplement: Figure S4 — In vitro stimulation of CD4+ T cells. (A) CD4 T cells were fractionated to Naïve, and LacZ+ and LacZ− effector memory T cells by sorting according to their expression of CD44 and CD62L. They were cultured with 2 µg/ml anti-mouse CD3e (clone 145-2C11, eBioscience) and 2 ug/ml anti-mouse CD28 (clone 37.51, eBioscience) for 2 Days. IL-2, IL-4, IL-17, IFN-γ and TNF-α in culture supernatants were measured by ELISA, Ready-SET-Go kit (eBioscience). The experiment was done twice and the results were essentially the same. (B) In vitro stimulation of Naive CD4+ cells failed to induce AID expression. Naive CD4+ T cells were sorted from Aicda-cre/R26R mice and cultured in vitro with various combination of stimulants with which T cells should polarize to different types of helper T cell states as indicated above each panel. The left most is the culture with un-fractionated CD4 T cells that included LacZ+ cells as a control culture. The data is the representative of two independent experiments. (TIF) [file pone.0029141.s004.tif]

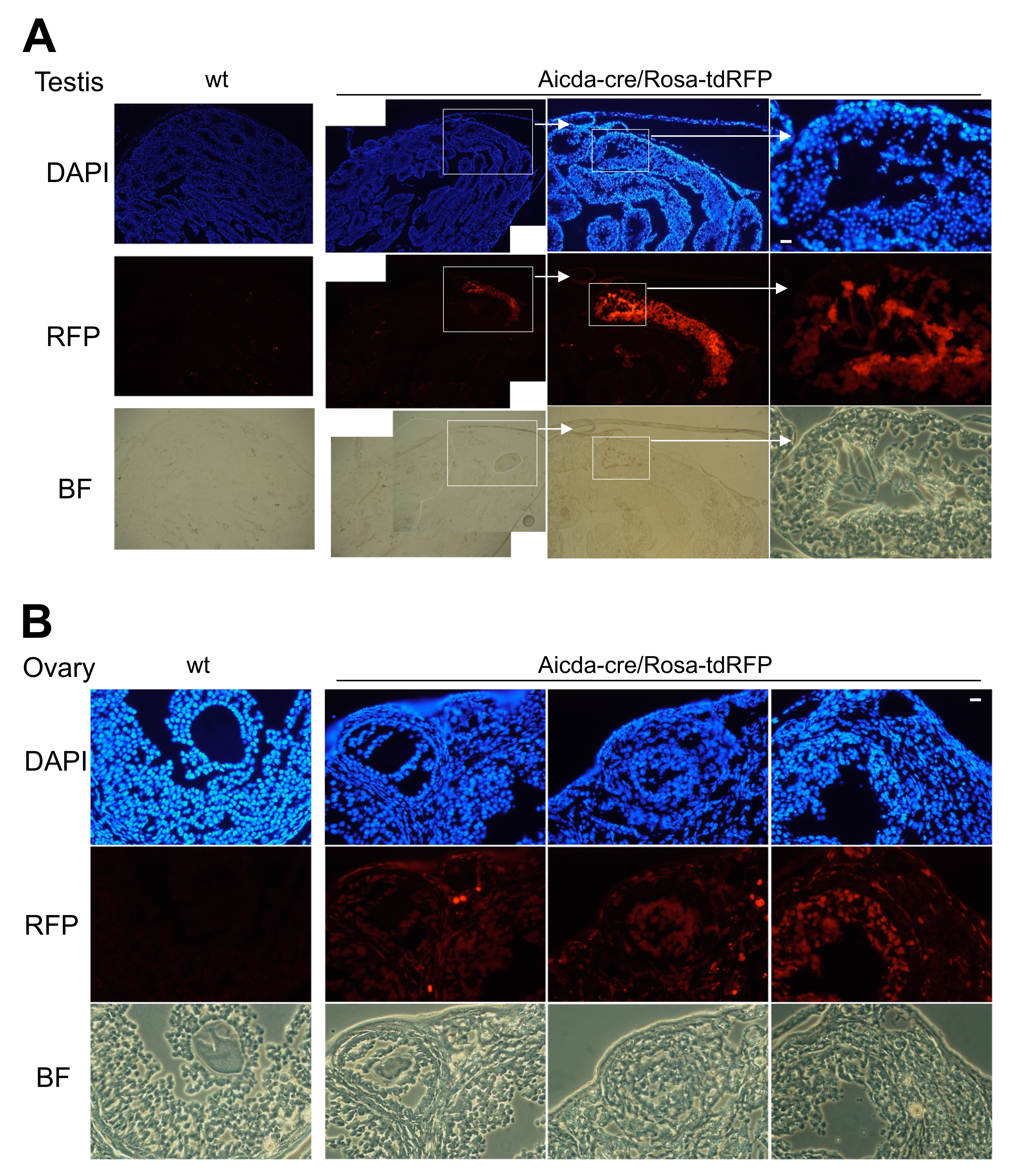

Supplement: Figure S5 — Histological analyses of testis and ovary. Frozen section of fixed tissues from Aicda-cre/Rosa-tdRFP and control mice (wt) were examined. Nuclear counter staining of DAPI and bright field image (BF) were shown below and above. (A) Images of testis. Boxed regions were magnified as indicated by arrows. A scale bar is shown in the high power magnification picture of the DAPI staining. Scale bar = 25 µm. (B) Images of ovary. Three different follicles of Aicda-cre/Rosa-tdRFP ovary are shown. Two sets of males and one set of female with the same number of controls were examined. (TIF) [file pone.0029141.s005.tif]
