# Supplementary material for: Activation-Induced Cytidine Deaminase Expression in CD4+ T Cells is Associated with a Unique IL-10-Producing Subset that Increases with Age
Source: PLoS One. 2011 Dec 28;6(12):e29141. doi: 10.1371/journal.pone.0029141 (PMC3247255; doi:10.1371/journal.pone.0029141)
Supplement: Table S1 — Mutation frequency of Myc exon 1-intron 1 region. (PDF) [file pone.0029141.s006.pdf]

Table S1. Mutation frequency of *Myc* exon 1-intron 1 region.

Mouse	Naïve T	Effector Memory T
wild-type	3/232,366<br>$0.13x10^{-4}$	10/232,297<br>$0.43x10^{-4}$
AID KO	5/259,491<br>$0.19x10^{-4}$	9/266,124<br>$0.34x10^{-4}$
