# Supplementary material for: Activation-Induced Cytidine Deaminase Expression in CD4+ T Cells is Associated with a Unique IL-10-Producing Subset that Increases with Age
Source: PLoS One. 2011 Dec 28;6(12):e29141. doi: 10.1371/journal.pone.0029141 (PMC3247255; doi:10.1371/journal.pone.0029141)
Supplement: Table S2 — Mutation analysis on randomly cloned TCR cDNA. (PDF) [file pone.0029141.s007.pdf]

Table S2. Mutation analysis on randomly cloned TCR cDNA.

	mutated/examined		frequency of mutation
	Clone #	base pair	/base pare
Naïve	5/102	5/26,796	$1.9x10^{-4}$
effector memory			
$LacZ^{-}hCD2^{-}$	2/77	2/19,958	$1.0x10^{-4}$
$LacZ^{+}hCD2^{-}$	5/85	5/22,404	$2.2x10^{-4}$
$LacZ^{+}hCD2^{+}$	7/124	8/34,426	$2.3x10^{-4}$
