# Supplementary material for: Activation-Induced Cytidine Deaminase Expression in CD4+ T Cells is Associated with a Unique IL-10-Producing Subset that Increases with Age
Source: PLoS One. 2011 Dec 28;6(12):e29141. doi: 10.1371/journal.pone.0029141 (PMC3247255; doi:10.1371/journal.pone.0029141)
Supplement: Table S3 — Primers used in this study. (PDF) [file pone.0029141.s008.pdf]

Table S3. Primers used.

Myc-F	5’-GCGTTTTTTTCTGACTCGCTGTAG-3’
Myc-R	5’-GCGGGGGTCAGGCTTAAATTTTACT-3’
Cα-1	5’-AGATTCCATGGTTTTCGGCAC-3’
Cβ-1	5’-GACCCCACTGTGGACCTCCTTGCC-3’
Cα-2-Xho	5’- AGACCGAGCTCGAGTTAACTGGTACAC-3’
Cβ-2-Xho	5’-TGCTTTTGACTCGAGAAACAAGGAGACCTTGGG-3’
anchor-Xba	5’-GGTCGACTCTAGAGGAGGCC-3’
anchor+dC-Xba	5’-GGTCGACTCTAGAGGAGGCCCCCCCCCCCCCC-3’
cyclophilin-F	5’- TGGAGAGCACCAAGACAGACA -3’
cyclophilin-R	5’- TGCCGGAGTCGACAATGAT-3’
